# Staphylococcus lugdunensis Endocarditis Causing Secondary Splenic Abscess: A Potentially Lethal Complication

**DOI:** 10.7759/cureus.52948

**Published:** 2024-01-25

**Authors:** Kishen Raj, Guo Hou Loo, Navindra Shamugam, Chee Loon Leong

**Affiliations:** 1 Department of Surgery, Vascular Surgery Unit, University Kebangsaan Malaysia Medical Center, Kuala Lumpur, MYS; 2 Department of Surgery, Upper Gastrointestinal and Metabolic Surgery Unit, University Kebangsaan Malaysia Medical Center, Kuala Lumpur, MYS; 3 Department of Surgery, Kuala Lumpur Hospital, Kuala Lumpur, MYS; 4 Department of Medicine, Kuala Lumpur Hospital, Kuala Lumpur, MYS

**Keywords:** infective endocarditis, targeted antibiotics, splenectomy, staphylococcus lugdunensis, splenic abscess

## Abstract

Infective endocarditis is a potentially life-threatening condition caused by a bacterial infection of the heart valves. The incidence of splenic abscess associated with infective endocarditis varies between 1-10% of cases, and its presence may indicate a severe form of the disease.

We present a 24-year-old man diagnosed with infective endocarditis who was found to have a splenic abscess upon further evaluation. The patient was initially managed conservatively with targeted antibiotics, but after unsuccessful percutaneous drainage, a splenectomy was performed. The patient underwent mitral valve replacement surgery and made a good recovery.

The patient's case highlights the importance of considering a secondary abscess in the management of infective endocarditis. This complication can easily be missed and cause significant morbidity.

This case underscores the importance of early diagnosis and effective collaboration between various healthcare professionals to achieve the best possible outcome for patients with infective endocarditis and its associated complications.

## Introduction

Infective endocarditis is a serious cardiac condition characterized by the growth of microorganisms on the heart valves and surrounding tissue. This disease is known to have a wide range of complications, including embolic stroke, splenic abscess, and infected splenic artery aneurysm [1]. A splenic abscess is an uncommon manifestation of infective endocarditis and can be life-threatening if not managed properly [2]. The incidence of splenic abscess associated with infective endocarditis varies between 1-10% of cases, and its presence may indicate a severe form of the disease [3].

A type of coagulase-negative staphylococcus (CoNS), *Staphylococcus lugdunensis* (*S. lugdunensis*), is a commensal of the human skin. Similar to other CoNS, *S. lugdunensis* has the ability to produce biofilm, which enables it to grow on cardiac valves and bioprosthetic tissues [1]. Similar to *Staphylococcus aureus* (*S. aureus*), it is an aggressive pathogen that causes a more severe form of infective endocarditis with an ensuing poor clinical outcome and carries a high mortality rate [4]. It has been reported to account for 18% of infective endocarditis caused by CoNS, and oftentimes, it may be misidentified as *S. aureus* [5].

Infective endocarditis of left-sided cardiac valves may be complicated by systemic embolization in up to 50% of cases, and the spleen is the most common site affected. A myriad of complications may occur, such as splenic infarction, hemorrhage, abscess, and splenic rupture. If untreated, splenic abscesses may be fatal [6]. As the mortality rate can be more than 20%, infective endocarditis patients with splenic abscesses are usually treated with splenectomy. Other options of treatment that have been described include percutaneous drainage as well as intravenous antibiotics alone [6].

With this in mind, we would like to report a young adult with *S. lugdunensis* an infectious endocarditis complicated by a large splenic abscess that did not respond to intravenous antibiotics alone. He subsequently underwent a splenectomy and a mitral valve replacement and made a good recovery. This work has been reported in line with the Surgical Case Report criteria [7].

## Case presentation

A 24-year-old male patient was admitted to the hospital with a complaint of fever and general fatigue. The patient had no prior medical history and maintained good oral hygiene with regular visits to the dental clinic. Upon admission, a pansystolic murmur was detected, and laboratory results showed a raised white blood cell count of 15.0 x 109/L and an elevated C-reactive protein level of 154 mg/L. An urgent echocardiogram revealed vegetation on the mitral valve, and a peripheral blood culture identified *S. lugdenesis*, leading to a diagnosis of infective endocarditis.

The patient was initiated on a regimen of intravenous cloxacillin after reviewing the culture sensitivity results, which showed signs of improvement. An abdominal ultrasound was performed to monitor the patient's bacteremia and revealed a 3 x 5 cm abscess in the spleen. The abscess was managed conservatively with targeted antibiotics, with plans for further ultrasound evaluation to assess the response. The patient was then transferred to a cardiovascular facility for valve replacement surgery.

**Figure 1:**
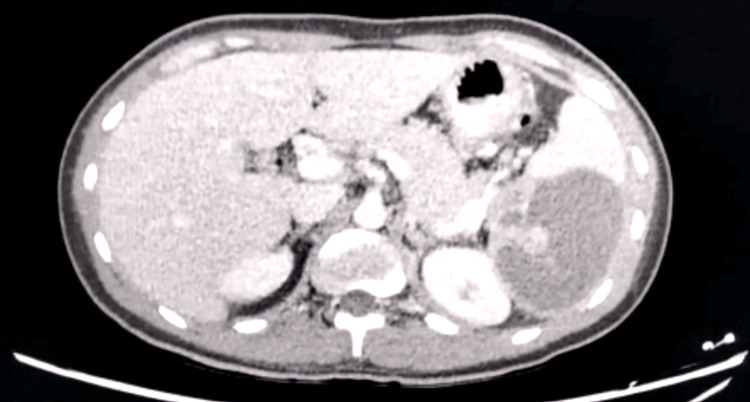
Axial view of the computed tomography (CT) abdomen showing a uniloculated hypodense lesion at the lower to mid-pole of the spleen measuring 10 x 12 cm

A follow-up echocardiogram showed further vegetation on the anterior aspect of the mitral valve, and a repeat ultrasound showed a larger abscess of 8 x 10 cm in the spleen. A contrast-enhanced CT scan was performed, which showed a unilocular hypodense lesion measuring 10 x 12 cm at the lower pole of the spleen (Figure 1). A spleen-preserving strategy was initially considered, and image-guided percutaneous drainage of the abscess was attempted, but it proved to be unsuccessful, and the patient did not respond to the conservative approach.

**Figure 2:**
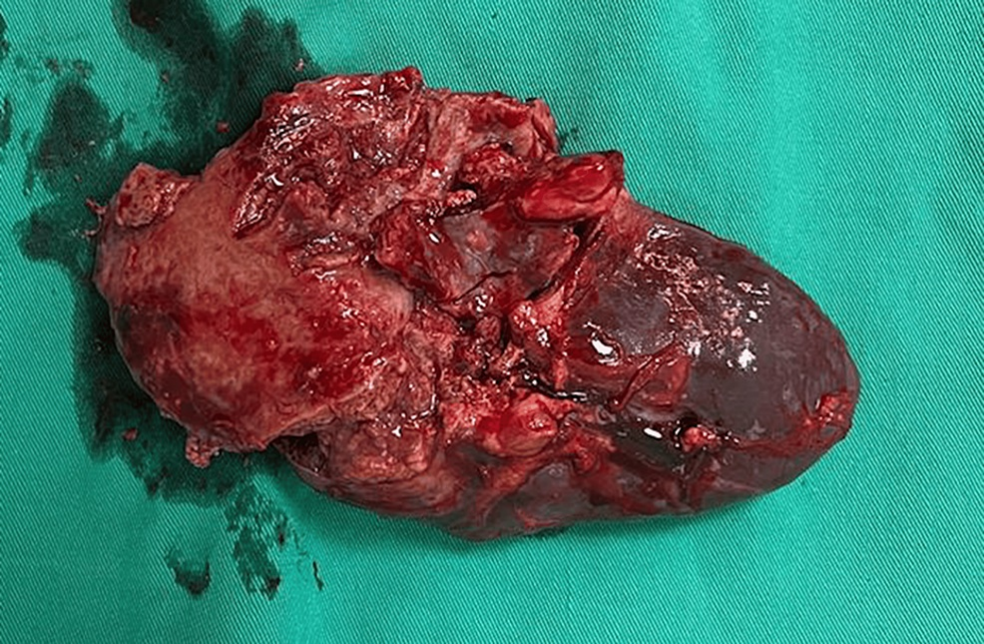
Splenectomy specimen showing a fragmented spleen with areas of necrosis

After consultation with a multidisciplinary team of cardiovascular surgeons, cardiologists, general surgeons, and infectious disease specialists, a decision was made to proceed with a splenectomy via a laparotomy approach. The splenectomy specimen was sent for histopathological and microbiological examination (which was consistent with *S. lugenesis*), and antibiotics were adjusted based on the sensitivity results (Figure 2). The patient received a combination of intravenous cloxacillin for 52 days, intravenous cefazolin for 24 days, and oral linezolid for 20 days.

The histopathological examination revealed an abscess cavity in the spleen. The patient underwent a mitral valve replacement surgery two weeks after the splenectomy, following a period of targeted intravenous antibiotics. The patient made a good recovery and was discharged one week after the surgery.

## Discussion

Infective endocarditis is a life-threatening condition that occurs when bacteria colonize heart valve surfaces, leading to valve damage and bacterial seeding into the bloodstream. Early diagnosis and prompt treatment are crucial to prevent serious complications such as septicemia, embolization, and valve damage. *S. lugdunensis* is a rare but important cause of endocarditis, with a reported incidence of 1-5% of all staphylococcal endocarditis cases [4]. Although it is a less common cause of infective endocarditis compared to other species of *Staphylococcus*, it is an aggressive pathogen that causes a more severe form of infective endocarditis with ensuing poor clinical outcomes and carries high mortality [4].

The patient, in this case, was diagnosed with both infective endocarditis and *S. lugdunensis* and a secondary splenic abscess, which is a rare but known complication of the condition [8]. In 20-50% of cases of infective endocarditis of the left-sided heart valves, systemic embolization occurs, with the spleen being the most commonly affected organ. This will lead to the formation of a splenic hemorrhage, splenic infarct, splenic rupture, or splenic abscess [6]. Splenic abscess likely originates from splenic infarction, and subsequently, either due to bacteremia or infected vegetation seeding the infarcted area, forms an abscess [6]. This case highlights the importance of considering a secondary abscess in the management of infective endocarditis, even without signs and symptoms of intra-abdominal infection such as left upper quadrant tenderness. This complication can easily be missed and cause significant morbidity. The presence of a secondary abscess in infective endocarditis patients can also have a significant impact on treatment outcomes, as the abscess can act as a reservoir for bacteria that may be resistant to antibiotics [6].

The clinical presentation of a splenic abscess in a patient with infective endocarditis is variable and may be non-specific. The most common finding is persistent fever, despite targeted antimicrobial therapy [8]. Other symptoms include left flank or left upper quadrant discomfort or pain, vomiting, hiccups, and abdominal distension. A left-sided pleural effusion or lower lobe infiltrate may be seen on a plain chest radiograph [8]. Prompt imaging of the abdominal cavity should be performed in a patient with the above findings, as splenic abscess carries a high morbidity and mortality rate [8].

The imaging of choice to diagnose splenic abscess is an abdominal CT scan, due to its high sensitivity and specificity [9]. A splenic abscess appears as a focal lesion of low attenuation with peripheral contrast enhancement after administration of intravenous contrast. In cases where it is difficult to differentiate a splenic infarct from an abscess, there is a role for image-guided percutaneous aspiration [9]. In our case, we elected to use an ultrasonography of the abdomen as it was readily available, and only when the splenic abscess failed to respond to the targeted antibiotics did we obtain a CT for surgical planning.

Splenectomy is the definitive management in patients with infective endocarditis complicated by splenic abscess, although intravenous antibiotics alone have been described in the literature [6]. In patients who are high-risk surgical candidates, there is a role for image-guided percutaneous drainage of the splenic abscess [8-9]. As there is a risk of secondary valve infection, valve replacement is usually performed after the splenectomy, although single-stage procedures have been described [8]. The laparoscopic approach, in experienced hands, offers the patient a faster recovery and lower morbidity when compared to the laparotomy approach [10]. Targeted antibiotic treatment should go hand in hand with surgery in the treatment of splenic abscesses [8]. The potential benefit of using a combination of antibiotics for the treatment of infective endocarditis is that multiple antibiotics may have a greater chance of success in eradicating the infection. Pneumococcal, meningococcal, and *Haemophilus influenzae* type B vaccinations should be given postoperatively to mitigate the risk of overwhelming post-splenectomy sepsis.

The patient's case also highlights the importance of a multidisciplinary approach in determining the best course of treatment for infective endocarditis patients. In this case, a team of cardiologists, infectious disease specialists, and surgeons worked together to diagnose the patient's condition and determine the most appropriate course of treatment [8]. This collaboration allowed for an optimal outcome, as the team was able to take into account the patient's overall health and medical history when making treatment decisions.

## Conclusions

This case highlights the potential for splenic abscess to develop as a complication of infective endocarditis and the importance of careful monitoring and multidisciplinary management. The presence of a splenic abscess in a patient with infective endocarditis mandates splenectomy, which should be performed before the valve replacement. Laparoscopic splenectomy in a stable patient may offer a better outcome in experienced hands.
